# Physical and Physiological Consequences of Babywearing on the Babywearer: A Systematic Review

**DOI:** 10.3390/healthcare13172193

**Published:** 2025-09-02

**Authors:** Yaiza Taboada-Iglesias, Andrés Domínguez-Estévez, Clara Rodríguez-Gude, Águeda Gutiérrez-Sánchez

**Affiliations:** 1 Special Didactics Department, Faculty of Science Education and Sport, Universidade de Vigo, 36005 Pontevedra, Spain; yaitaboada@uvigo.es (Y.T.-I.); agyra@uvigo.gal (Á.G.-S.); 2 Education, Physical Activity and Health Research Group (Gies10-DE3), Galicia Sur Health Research Institute (IIS), Universidade de Vigo, 36005 Pontevedra, Spain; 3 Graduate in Physical Activity and Sport Sciences, Universidade de Vigo, 36005 Pontevedra, Spain; dominguezandrez10@gmail.com; 4 Functional Biology and Health Sciences Department, Faculty of Physiotherapy, Universidade de Vigo, 36005 Pontevedra, Spain

**Keywords:** baby carrier, energy expenditure, ergonomic carrying, muscle activation, posture

## Abstract

**Background/Objectives**: Babywearing is a carrying system that ensures consistent contact and proper posture between the baby and carrying adult, in which there is no age or weight limit, and it is rarely inadvisable. Although babywearing has been growing in popularity and acclaim due to the comfort and emotional closeness between the carrier and baby, there are a number of physical and physiological consequences for the adult carrier when using an ergonomic babywearing device, such as muscular, postural, cardiorespiratory, and energy expenditure, and the perception of effort and pain. The objective is to explore the physical implications affecting the carrier, as well as the subjective perception of strain and pain. **Methods**: A systematic review was carried out including articles up to December 2023 in the Web of Science (WOS), Medline, and SportDiscus databases. Studies dealing with ergonomic babywearing and the physical implications of babywearing were included; systematic reviews or case studies were excluded. **Results**: After applying the inclusion and exclusion criteria, a total of 14 original articles were obtained for analysis. Methodological quality was rated using the Joanna Briggs Institute Critical Appraisal Checklist for Analytical Cross-Sectional Studies with scores between 3 and 8 points. All articles included valid and reliable information on exposure, outcome measures, and results. **Conclusions**: The studies reviewed cover different aspects, such as muscle activation and postural stability, as well as specific ergonomic design for particular groups. In general terms, it seems that the use of certain babywearing devices, especially back or front carry, seems to be the one that generates fewer physiological alterations in the carriers compared to carrying babies in arms or other positions.

## 1. Introduction

The use of babywearing devices is spreading in the population, so much so that it has gained popularity and has experienced a remarkable increase in recent years, being a beneficial practice due to the constant contact between the baby and carrying adult [1]. Furthermore, appropriate babywearing has the potential to consistently support infants’ spines [2]. This carrying practice involves carrying the baby in an ergonomically designed baby carrier, in which the baby’s weight is evenly distributed. Although babywearing has been growing in popularity and praise due to the comfort and emotional closeness between the carrier and baby, there are still some precise questions and doubts about the physical and physiological implications for both the baby and adult. Furthermore, there are certain aspects that pediatricians and health professionals should inform in a correct manner and encourage good practice, as it is often not known what the appropriate devices are, whether it is conducted safely or not, and what the benefits are [3].

Babywearing can also involve differentiated strain between different types and methods of carrying. In the study by Fista et al. [4], subjective strain perception was measured using the BORG scale, in which the babywearer used three different types of baby carriers (a soft structured carrier, a ring sling, and a traditional shawl). The data indicated that the mechanism that caused the most strain was the baby carrier with a ring on the left shoulder because the sling rings made of metal put pressure on the left shoulder. In addition, they found that it also affected other areas such as the neck, back, and trunk.

The physical load is an important consideration to take into account, as it generates different muscle activations in the babywearer. Certain baby carriers generate less activation in the spinal muscles compared to other situations and therefore generate less injurious strains [5]. Yuk et al. [6], using electromyography (EMG), observed that different muscle patterns are activated depending on the method of carrying.

Just as there are muscle specificities of different physical activities, the practice of babywearing affects the adult’s posture and gait. According to Azaman et al. [7], the pelvis, head, and shoulder are affected by carrying. In addition, carrying in the arms increases the load-bearing knee abduction moment by 8.7% and the load-bearing knee extension moment by 16.7% [8].

Also, the technique and duration of carrying and the physical characteristics of the carrying adult can alter cardiorespiratory aspects, and the energy expenditure related to ergonomic carrying can affect endurance and the ability to perform other physical tasks during the day. There are differences in energy costs [9] and perceived effort [10] between carrying a baby in the arms versus carrying a baby in a carry system.

Furthermore, the occurrence of pain is an important issue to consider when deciding whether to babywear or not. In the study by Mannen et al. [11], pain perception was analyzed using the Visual Analogue Scale. The results indicated that carrying adults experience more pain when carrying babies than when babywearing devices are used. This seemed to be due to the fact that when babies are carried, the body is in an asymmetrical position, and there are more areas of swaying.

It is important to assess the impact of babywearing in order to take appropriate measures to improve physical fitness. The design of baby carriers and their technique of use should be studied to minimize the risk of injury due to poor posture or increased muscle fatigue from prolonged carrying.

From the above, it appears that babywearing offers numerous benefits to ensure a comfortable and safe experience for the adult and baby, but it also has physical implications that affect the babywearer, and knowing the alterations produced by this practice of carrying and care, it will be possible to design exercise programs adapted to these populations, focused on preventing injuries or improving the experience.

It is important to better understand the physical implications for the adult using babywearing devices in order to generate exercise programs that prevent possible alterations or injuries. In addition, it seems relevant to provide information to parents, carrying adults, health professionals, and product designers in order to promote safe and healthy babywearing practices that benefit both the baby and the carrying adult.

Therefore, the aim of this paper is to explore the physical implications affecting the babywearer, focusing on physical and physiological aspects such as muscle activity, posture, and gait, and cardiorespiratory and energetic factors, as well as subjective perception of effort and pain.

## 2. Materials and Methods

### 2.1. Databases and Searches

This systematic review aimed to answer the following: What are the effects of different babywearing methods on (a) muscle activation, (b) postural stability, (c) energy expenditure, and (d) subjective pain and fatigue in healthy adults?

The PRISMA 2020 statement (Preferred Reporting Items for Systematic reviews and MetaAnalyses) [12] was followed for the search of the articles included in this review. This review protocol was developed prior to data extraction and is publicly available at OSF: https://osf.io/qjp4r/ (accessed on 29 December 2023). The search was conducted in the following databases: Web of Science (WOS), Medline, and SportDiscus, including results up to December 2023.

The following PICO(S) strategy was followed: Patient (babywearers); intervention (physical and physiological implications); comparison (other carrying methods); outcome (effects of babywearing).

The search strategy was applied independently in each database following the particularities of each one. The Medical Subject Heading (MESH) terms used in the search were “Physical fitness” [Mesh], “Postural Balance” [Mesh] and “Biomechanical phenomena” [Mesh]. In addition, the keywords used to identify articles on the subject of this review were ‘baby carrier’, ‘ergonomic carrier’, ‘babywearing’, ‘baby carrying’, combined with ‘electromyography’, ‘muscle activity’, ‘postural stability’, ‘body balance’, ‘ergonomic’, ‘center of pressure’, ‘physical condition’, and ‘posture’. The Boolean operators OR and AND were used to link the terms and establish the search equations (Table 1).

All studies were assessed independently by two reviewers. Each reviewer screened titles and abstracts to obtain relevant full articles which were assessed against the inclusion and exclusion criteria described. In case of a lack of consensus, the judgment of a third reviewer prevailed.

### 2.2. Selection of Studies and Selection Criteria

For the selection of articles, studies dealing with babywearing and the physical implications of babywearing were set as inclusion criteria. Exclusion criteria were systematic reviews or case studies (Table 2).

**Figure 1:**
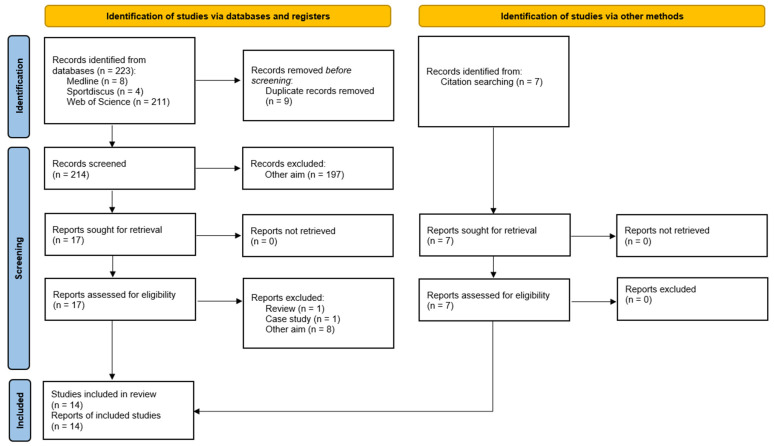
PRISMA flowchart of the item selection process.

The search identified 223 articles in the three databases, of which 9 were eliminated as duplicates. Of the 17 full-text articles assessed, 10 were excluded due to the fact that they were case studies, reviews, or articles that did not address the topic, resulting in 7 studies from the database search. Notably, 7 additional studies were identified through citation searching, yielding a total of 14 studies for inclusion. The entire process detailing the study selection steps can be found in the PRISMA flowchart (Figure 1).

### 2.3. Variables

This research includes data on the effects of babywearing on the babywearer’s body, including variables such as muscle activation, load distribution and pressure, skin temperature, heart rate, stability, posture, balance, pain, gait kinematics, volumes, and respiratory rate, among others. For the interpretation of the results, results with statistical significance were compared. When studies use different units of measurement, results shall be standardized as far as possible. In addition, if the results provided are by subgroups, these shall be taken into account in the analysis.

### 2.4. Assessment of Methodological Quality and Risk of Bias of Individual Studies

The Joanna Briggs Institute Critical Appraisal Checklist for Analytical Cross-Sectional Studies (JBI) scale for analytical cross-sectional studies was used for the analysis of methodological quality [13].

The JBI scale uses eight criteria to assess the overall methodological quality of a study. Criteria for inclusion: sample inclusion criteria; description of subjects and settings; valid and reliable exposure measure; objective and standard measure of condition; identification of confounders; strategies to address confounders; valid and reliable outcome measure; and adequate statistical analysis. The final score ranges from 0 to 8 points. There is no ranking criterion for study quality. For ease of interpretation, we classified the methodological quality score, regardless of the scale used, as low (0–3 points), medium (4–6 points), and high (>6 points), as was conducted in a previous study [14].

## 3. Results

Methodological quality analysis yielded scores ranging from 3 to 8 points. One article scored low [15], nine scored medium [4,5,6,7,8,9,16,17,18], and four scored high [11,19,20,21] (Table 3). All studies met criteria 3 (exposure was measured in a valid and reliable manner), 4 (objective and standardized criteria were used to measure the condition), and 7 (outcomes were measured in a valid and reliable manner).

Table 4 showed the different types of carrying evaluated in each of the studies, while Table 5 summarized the main results and research characteristics of the studies included in this review, grouped by the different variables studied. We found that variables such as muscle activation have been evaluated in four studies [5,6,18,21]. Posture or stability has been investigated in six of the studies [4,7,8,9,15,17], while foot pressure or center of pressure has been investigated in five [6,7,11,18,19]. Physiological variables such as heart rate, skin temperature, or respiratory values have been evaluated in four of the studies [4,16,20,21].

## 4. Discussion

This paper has analyzed the physical and physiological consequences of babywearing on the babywearer. To the authors’ knowledge, this is the first systematic review on this topic. The results analyzed in this review come from articles with acceptable methodological quality, with only one article rated with a low score on the JBI scale.

When interpreting the different results obtained in the studies, methodological differences have been observed, such as the use of dummies versus babies, the different walking or standing protocols used, as well as the different designs of carrying systems used, among others. Such heterogeneity in the studies has made direct comparability between them difficult.

### 4.1. Muscle Activity

Different types of baby carriers affect the muscle activation of the babywearer and in this case, EMG is a widely used tool. In the study by Fagundes et al. [5], an electromyographic analysis of the spinal muscles was performed during walking and with different baby carriers. In terms of the results, baby carriers that do not distribute the weight evenly generate more activation of the spinal muscles. In contrast, the kangaroo method activates these muscles the least compared to other situations. In the study by Yuk et al. [6], they also used EMG to study the activation of trunk and lower limb muscles such as the biceps femoris. Their results show that front babywearing (LBC-F) generates greater activation in all the muscles analyzed than back babywearing (LBC-B), which could result in greater muscle fatigue and injury with prolonged use. On the other hand, in a high-quality study [21], the activation of the upper trapezius was greater when carrying a two-part front-facing backpack than with a front-facing backpack with shoulder straps. In addition, upper trapezius and erector spinae activation was statistically significantly higher in males and also in heavier babies, while rectus abdominis activation was higher in females. Based on this, an ergonomic design that minimizes muscle loading and favors adequate weight distribution of the baby is important.

### 4.2. Posture and Gait

The use of baby carriers has a significant impact on the babywearer’s posture and gait. Azaman et al. [7] found that baby carriers can alter the wearer’s posture, increasing postural sway. Thus, according to the results analyzed, the pelvis, head, and trunk are affected by the load. Another curious fact is that carrying a baby carrier in the front produces less anteroposterior deviation of the joints than back or side baby carriers.

Along the same lines, Havens et al. [17] analyze how different methods of carrying the baby affect the carrier’s posture during walking and picking up objects. Thus, carrying the baby with unequal weight and asymmetrical posture results in back extension, which affects the efficiency of movement. In terms of retrieval, most individuals performed the squat technique to retrieve the object from the floor, both while carrying it and with it in their arms.

And for its relationship with lower and upper limbs, the study by Williams et al. [8] also examines the babywearer’s posture using different infant carrier methods, and the resulting data are similar to the previous study in that carrying in arms increases the carrying knee abduction moment by 8.7% and the carrying knee extension moment by 16.7%. Hyun and Ryew [19] studied the effects of carrying an infant using a rear carrier during high-heeled walking in high-quality research. After analyzing the 1st and 2nd peak vertical force (PVF), there are statistically significant differences according to the use of high heels and walking conditions. On the other hand, medial-lateral center of pressure (COP) and leg stiffness showed statistically significant differences according to heel height and walking conditions. This finding is particularly relevant for babywearers performing activities where posture and stability are crucial, such as walking on inclined or uneven surfaces.

### 4.3. Load

Another factor to consider is the weight of the baby. Chen et al. [16] investigated the maximum acceptable load during babywearing, finding that men carried more weight than women, that more weight could be carried in 1 h than in 4 h, that when back babywearing, the acceptable weight was greater than when front carrying, and that backpacks with shoulder straps carried more weight than two-part backpacks. These data will be important for babywearers to consider, as depending on the weight of the baby, the choice of both baby carrier and carrying position may vary. In addition, the length of use should also be considered, as excessive use can be harmful even with regard to the recommended weight for each device.

### 4.4. Cardiorespiratory Effects

The impact of baby carrier use on cardiorespiratory response and energy expenditure is another important determinant to consider. When assessing heart rate (HR), Wu et al. [21] found, in their high-quality study, that HR was statistically higher in women, and that the weight of the baby also affected exercise intensity, as it was higher when carrying a 10 kg baby than a 7 kg baby. Chen et al. [16] also assessed HR, finding that the percentage of maximum heart rate (%HR) was higher when carrying for 1 h than for 4 h. A study evaluating the relationship between the HR of babywearers and babies during babywearing also found that babywearing increases the babywearer’s HR, but the baby’s HR decreases, especially when the mother is carrying the baby [22].

In the high-quality study by Ohashi et al. [20], the respiratory response during downhill walking was analyzed by front and back carrying with baby carriers, and in both situations, the respiratory effort increased significantly. This effort is accompanied by higher energy expenditure, which leads to lower locomotion efficiency and greater fatigue during prolonged use. From a different perspective, the study by Wall-Scheffler et al. [9] analyzed the effects on the musculoskeletal system of carrying a baby using different carrying mechanisms. The results indicated that there is an average increase in the perceived physical effort when carrying a baby in arms compared to carrying a baby in a sling (16%, ranging from a 13% to a 25% increase). In addition, standardized stride lengths are shorter during carriage in arms than when using a front carrier. Following these explorations, it is of vital importance to design babywearing devices that promote ergonomic posture, reduce energy expenditure, and improve movement efficiency.

### 4.5. Fatigue and Pain

To assess comfort and fatigue during babywearing, perceived exertion can help us, even though it is a subjective measure. In the study by Chen et al. [16], front carrying had a higher value in perceived exertion compared to back carrying, both in the lower back and abdomen, as measured by the Borg CR-10 scale. Fista et al. [4] used a physiological and biomechanical approach to evaluate different methods of use (Soft Structured Carrier (SSC) harness, Jarik, and ring sling) of baby carriers. The data indicated that the mechanism that caused the most strain was the ring sling and the Jarik, especially on the left shoulder. The SSC-type harness provided the best change of posture and the highest level of confidence. Similarly, Lee and Hong [18] measured muscle fatigue and COP in different types of baby carriers. They found that designs that allow a more natural posture and distribute weight evenly reduce muscle fatigue. This reduction in muscle fatigue translates into less perceived strain on the part of the carrier, which is crucial for carrier acceptance and prolonged use, since the time spent carrying babies is considerable [23].

As with the previous variable, pain perception is also important to take into account when carrying and is a good indicator of whether the ergonomic design is adequate or not. To this end, the study by Mannen et al. [11], considered to be of good quality, investigated how different methods of carrying the baby (unloaded, carrying a dummy in arms, carrying a dummy) affect postural sway and pain during prolonged standing. The results indicated that 30% of the participants reported pain during unloading and carrying, while 50% reported pain during carrying. Participants shifted their weight more frequently as they spent more time in an asymmetrical position and had greater areas of swinging in weight bearing and therefore increased the perception of pain, especially in the lumbar region. In relation to the previous article and the pain that can be suffered in the back while carrying, one study [15], which had moderate methodological quality, suggested the need to design baby carriers that provide good lumbar support and distribute the weight in a balanced way, especially for older people, as this can reduce pain in the back and joints. Another factor pointed out by these authors is the posture of the shoulders and hips while using the baby carrier by the users.

### 4.6. Limitations, Future Lines of Research, and Practical Implications

In terms of limitations, while the chosen databases are major sources, a broader search might have captured additional relevant literature. The review includes studies with different experimental designs and measurement methods, which may limit the comparability of results. On the other hand, most of the studies focus on young and middle-aged adults, and most of them are conducted only on women, which makes it difficult to extrapolate results to the male or older population; however, the profile found in the studies corresponds to the population that tends to use these parenting aids the most. Furthermore, the interpretation of some of the variables studied, such as heart rate or muscle activation, may imply a difference in results depending on the characteristics of the wearer, including age, sex, and anthropometry. A number of studies are conducted in controlled settings and with dummies simulating babies rather than real children, which may not reflect the conditions of real, everyday use.

In terms of future lines of research, the long-term effects of baby carrier use on the health of the carriers in real, everyday conditions could be investigated, as well as extending studies to broader groups such as older people, individuals with disabilities, or different ethnicities. In addition, research can be conducted on the incorporation of new smart technologies in baby carrier design.

Furthermore, this information can serve as a basis for designers to improve the design of baby carriers, making them more ergonomic and adaptable to different carriers. As for health professionals, they should use this information to educate users about the best types of baby carriers and the importance of good posture. In addition, these experts should apply the knowledge to develop specific exercise and stretching programs for babywearers. As for parents, understanding the effects of different types of baby carriers helps them choose which one to use, given the wide variety available on the market.

## 5. Conclusions

The analysis carried out on the existing literature on the use of baby carriers and their relationship to carrier ergonomics reveals a number of important findings that should be considered by both health professionals and baby carrier designers. The studies under analysis cover different aspects, such as muscle activation and postural stability, as well as specific ergonomic design for particular groups. The synthesized evidence suggests that back carriers seem to be the least likely to cause physiological alterations in babywearers compared to babywearing babies in arms or other positions.

On the one hand, it has been shown that different ways of carrying the baby (front, back, or in arms) affect the electromyographic activity of the spinal muscles, and, in terms of muscle fatigue, it is essential that baby carriers minimize excessive activation of the trunk and lower limb muscles to reduce muscle fatigue and the risk of injury, as this can have a considerable impact on the user’s musculoskeletal health in the long term, with kangaroo or back carry mode being found to generate the least activation.

On the other hand, depending on the type of baby carrier, these can influence the wearer’s posture and gait, so a design that favors even weight distribution and supports a natural posture is crucial to maintain stability and gait efficiency.

Infant carrying methods also affect breathing and energy expenditure compared to carrying the baby in your arms, so the design should be optimized to reduce respiratory and energy expenditure, which can significantly improve the comfort and prolonged wearability of the baby carrier. Therefore, in addition to design factors and carrying modalities that minimize injury and improve the posture and stability of the carrier, intervention through physical exercise is important to reduce and prevent physical problems that may affect the carriers. It is also important to take into account the duration of use due to the load being carried, trying not to exceed the time limit, and taking breaks whenever possible to avoid injuries due to overexertion.

## Figures and Tables

**Table 1:** Search equations from different databases.

DB	Search Equation
MEDLINE	(“baby carrier” OR “ergonomic carrier” OR “Baby carrying” OR “Babywearing”) AND (“muscle activity” OR “electromyography” OR “postural stability” OR “Ergonomic” OR “center of pressure” OR “body balance” OR “physical condition” OR “Physical fitness” OR “Postural Balance” OR “Biomechanical phenomena” OR “Posture”)
SPORTDISCUS	(“baby carrier” OR “ergonomic carrier” OR “Baby carrying” OR “Babywearing”) AND (“muscle activity” OR “electromyography” OR “postural stability” OR “Ergonomic” OR “center of pressure” OR “body balance” OR “physical condition” OR “Physical fitness” OR “Postural Balance” OR “Biomechanical phenomena” OR “Posture”)
WEB OF SCIENCE	(((ALL = (baby carrier)) OR ALL = (ergonomic carrier)) OR ALL = (baby carrying)) AND (((((((((((ALL = (muscle activity)) OR ALL = (electromyography)) OR ALL = (postural stability)) OR ALL = (ergonomic)) OR ALL = (center of pressure)) OR ALL = (body balance)) OR ALL = (physical condition)) OR ALL = (physical fitness)) OR ALL = (Postural Balance)) OR ALL = (biomechanical phenomena)) OR ALL = (posture))

DB: databases.

**Table 2:** Inclusion and exclusion criteria.

Inclusion Criteria	Exclusion Criteria
- Studies dealing with babywearing - Physical or physiological implications for the adult carrying the baby (muscular voluntary contraction, center of pressure, heart rate, visual analogue scale, etc.).	- Systematic reviews - Case studies

**Table 3:** Methodological quality scores of the “Joanna Briggs Institute Critical Appraisal Checklist for Analytical Cross-Sectional Studies”.

Author (Year of Publication)	1	2	3	4	5	6	7	8	Score *
Wu et al. (2017) [21]	+	+	+	+	−	+	+	+	7
Chen et al. (2019) [16]	−	+	+	+	−	+	+	+	6
Azaman et al. (2017) [7]	+	+	+	+	−	−	+	+	6
Lee & Hong (2018) [18]	+	−	+	+	−	−	+	+	5
Wall-Scheffler et al. (2007) [9]	−	+	+	+	−	−	+	−	5
Havens et al. (2020) [17]	+	+	+	+	−	−	+	+	6
Ohashi et al. (2018) [20]	+	−	+	+	+	+	+	+	7
Mannen et al. (2020) [11]	+	+	+	+	+	+	+	+	8
Fista et al. (2019) [4]	−	+	+	+	−	−	+	−	4
Williams et al. (2019) [8]	+	+	+	+	−	−	+	+	6
Yuk et al. (2010) [6]	+	+	+	+	−	−	+	+	6
Hyun & Ryew (2018) [19]	−	+	+	+	+	+	+	+	7
Fagundes et al. (2016) [5]	+	+	+	+	−	−	+	+	6
Atthawuttikul & Khongkharat (2021) [15]	−	−	+	+	−	−	+	−	3

1. Were the criteria for inclusion in the sample clearly defined? 2. Were the study subjects and the setting described in detail? 3. Was the exposure measured in a valid and reliable way? 4. Were objective, standard criteria used for measurement of the condition? 5. Were confounding factors identified? 6. Were strategies to deal with confounding factors stated? 7. Were the outcomes measured in a valid and reliable way? 8. Was appropriate statistical analysis used? +: Yes; −: No. * The colors represent the different scores: red for low (0–3 points), orange for medium (4–6 points), and green for high (>6 points).

**Table 4:** Types of carrying evaluated in each of the included studies.

	Load Type	Frontal Load		Back Load		Side Load	Carry	No	Figure
	(kg)	Backpack	Sling	Backpack	Sling	Sling	in arms	load	included
Wu et al. (2017) [21]	D (7 & 10)	X *	X						X
Chen et al. (2019) [16]	D (up to 7)	X *		X *					X
Azaman et al. (2017) [7]	D (5)		X		X	X	X	X	X
Lee & Hong (2018) [18]	D (7.6)	X *							X
Wall-Scheffler et al. (2007) [9]	D (7.6)				X		X		
Havens et al. (2020) [17]	D (5)	X					X	X	X
Ohashi et al. (2018) [20]	D (8.4)	X		X					X
Mannen et al. (2020) [11]	D (6 m)	X					X	X	X
Fista et al. (2019) [4]	D (7.3)	X	X *						X
Williams et al. (2019) [8]	D (2.7)	X					X	X	X
Yuk et al. (2010) [6]	D (7.6)	X	X	X				X	X
Hyun & Ryew (2018) [19]	B (<1 y, mean 10.9)			X				X	X
Fagundes et al. (2016) [5]	D (6)	X	X				X	X	X
Atthawuttikul & Khongkharat (2021) [15]	S	X *							X

* Indicates that the same study evaluated different baby carriers within the same type of load or subvariations within the same carrying device (e.g., tight vs. loose fit); B: baby; D: dummy; m: months; S: load simulated with software; y: year old; X: indicates the types of carrying included in each investigation.

**Table 5:** Summary of studies included in the review.

Author (Year)	Sample	Scope/Variables Analyzed	Intervention/Methods	Results
Fagundes et al. (2016) [5]	N = 20 ♀ healthy right-handed women without previous births (23.4 ± 1.39 years)	-Program EMG Lab V1.1. (lower trapezius and lumbar spinal erectors)	-The women walked for 3 min without load at a speed of 6 km/h. -In each baby-carrying situation, data were recorded for 60 s. -Collections were performed: (A) With the baby in a horizontal position, with the head facing the right side. (B) With the baby on your lap in an upright position. (C) Using kangaroo baby carrier, with arms alongside the body. (D) Using a sling (hammock) with the volunteer’s arms alongside the body. (E) No load (Control).	EMG in -Load shape [F_(4, 59)_ = 17.1 *p* < 0.001] and side [F_(1, 59)_ = 89.6 *p* < 0.001] affected the intensity of erector spinae muscle activity. -Load shape [F_(4, 59)_ = 6.4 *p* < 0.001] and side [F_(1, 59)_ = 59.9 *p* < 0.001] affected the intensity of trapezius muscle descending fiber activity. -The trapezius obtained greater activation (*p* < 0.001) in form (a) than in forms (b), (c), and (e). -The left side, in all forms of loading, had the same intensity of activation of the trapezius muscle. -On the right side, form (a) showed greater activation than forms (e), (d), and (b). -EMG activity in the erector spinae muscle in form (b) was higher than in forms (a), (c), and (e). -Carrying form (d) showed greater activation than forms (c) and (e). The kangaroo carrier form generated less activation in the spinal muscles compared to other situations.
Yuk et al. (2010) [6]	N = 31 ♀ (23.2 ± 2.39 years)	-EMG (internal oblique, T4, L3, and L5 paravertebral, vastus medialis, biceps femoris, tibialis anterior, and gastrocnemius). -MATSacnSystem pressure foot platform.	Standing in all 4 positions: (A) Standing without load (SWC) (B) Carrying in front (LBC-F) (C) Carrying behind (LBC-B) (D) In shoulder carry (SC)	EMG: -Significant differences were found in the activation of biceps femoris (higher in LCB-F and lower in LCB-B), T4 (higher in LCB-F and LCB-B), L3 (higher in LCB-F, lower in LCB-B), L5 (higher in LCB-F, lower in LCB-B), and paravertebral (*p* < 0.05). -The other muscles did not show significant differences Foot pressure platform: -The pressure platform in the three forms of carrying obtained significant differences in the medial area of the right foot with respect to SWC.
Wu et al. (2017) [21]	N = 10 women (27.6 ± 3.8) and 10 men (27.8 ± 4.5)	-EMG (right side muscles: upper trapezius, rectus abdominis, erector spinae). -Skin temp. (upper back: midpoint between C7 and acromion; abdomen: 3 cm above the umbilicus). Shoulder pressure distribution. Heart rate.	The intervention was carried out on two different days. There were 6 combinations (3 types of baby carriers and 2 weights). In each combination, the baby was carried walking at a comfortable speed for 20 min while moving the arms freely. A 10 min break was taken between each combination.	EMG: -Upper trapezius and upper erector spinae in males (*p* < 0.001, *p* = 0.047, respectively), and rectus abdominis was significantly greater in females (*p* < 0.001). -Baby weight: upper trapezius and erector spinae were significantly greater when carrying a 10 kg baby than when carrying a 7 kg baby (*p* < 0.001). -Upper trapezius was significantly affected by baby carrier type: lower when using baby carrier type A than when using baby carrier type B, while baby carrier type C produced a similar response to the other two carriers. Body skin temperature: -Abdominal skin temp was higher in females (*p* < 0.05). -Carrier effect: Upper back temp was significantly different among the three carriers (*p* < 0.001). Type B produced the highest upper back skin temperature, while the use of type A and C carriers produced a lower upper back skin temperature. -Effect of weight: carrying a 7 kg baby resulted in higher abdominal skin temp (*p* < 0.001). Shoulder pressure distribution: -Significant differences according to baby carrier in shoulder pressure (*p* < 0.05) and maximum shoulder pressure (*p* < 0.001): type A produced the highest shoulder pressure and the highest maximum shoulder pressure, while baby carrier types B and C produced lower shoulder pressure, and baby carrier type B produced the lowest maximum shoulder pressure. Baby weight: the heavier the baby, the greater the pressure and the greater the maximum shoulder pressure. Heart rate: -Mean HR was higher in females (*p* < 0.05). -Baby weight: exercise intensity was greater with a 10 kg baby (*p* < 0.05).
Lee & Hong (2018) [18]	Healthy ♀ between 26 and 39 years	-COP: Foot pressure -Stability, comfort, and subjective fatigue -EMG: % of MVC	The following variables were measured using 3 types of baby carriers (X-type, H-type, and H-hip-type) and in two positions (fitted and loose). At rest and after walking for 30 min (with 30 min rest between carrier types). -COP: Body pressure measuring device (60 frames/s for 2 min). -EMG: Neck extensors, lower trapezius, thoracic and lumbar spinal erectors, rectus femoris, biceps femoris, tibialis anterior, medial calf. -Stability, comfort (shoulders, chest, and waist), and subjective fatigue: After 30 min walking (11-point Likert scale and Borg’s CR-10 scale).	Stability: -The babywearers showed differences in medial/lateral shifts (*p* < 0.05), and the babywearing devices showed significant antero/posterior and diagonal differences (*p* < 0.05). -Antero/posterior displacement was greater in the H-type than in the H-hip type, with the H-type showing less stability than the other two. -Changes in COP, COP displacement, COP velocity, and anterior/posterior movement increased when baby carriers were used loosely compared to when they were used tightly (*p* < 0.01). Muscle fatigue: -Carrying types and mode only had significant interaction with fatigue in the medial gastrocnemius (*p* < 0.01). -Among the baby carrier types, X-type generated more muscle fatigue in the lower trapezius, thoracic spinal erectors, and biceps femoris than the other types. -The manner of use also resulted in differences in muscle fatigue. Subjective fatigue: -The center of mass shifted downwards when the carriers were worn loose, increasing pressure on the waist and femoral region. Comfort: -At the shoulders, it is more satisfactory when baby carriers are worn snug (*p* < 0.01), but comfort at the chest is better when loose (*p* < 0.01). -Waist comfort is better in the X-type (*p* < 0.01). They conclude that subjective wearing comfort worsens the more the body is covered, wrapped, and pressed, regardless of postural stability or muscle fatigue.
Azaman et al. (2017) [7]	N = 15 healthy ♀ (23.1 ± 1.03 years)	-COP: Distribution of M and Force in x, y, z axes. -Posture: Joint displacement (3D ROM of the markers located at the head, shoulders, pelvis, hip, knee, and ankle).	-All participants were measured under the following conditions: (A) No load (control) (B) Loaded (L) (C) Loaded with baby carrier in front (LBC-F) (D) Loaded with baby carrier on the side (LBC-S) (F) Loaded with baby carrier on the back (LBC-B) -They stood for 1 min on the platform and rested for 5 min between postures.	COP: -Anterior displacement in L and LBC-F relative to control (*p* < 0.05). -LBC-F appears to displace the COP less than without baby carrier, but not significantly so. -LBC-F produces less anterior-posterior displacement and more lateral displacement than LBC-S or LBC-B but not significant (*p* > 0.05). Posture: -Pelvis, head, and shoulder are affected by load. -LBC-F produces less anteroposterior deviation of the joints than the other baby carrier situations.
Williams et al. (2019) [8]	N = 18 healthy ♀ (22.67 ± 2.08 years)	-Posture: camera and reflective markers	-3 walking sessions under the following conditions: (A) A baseline of three minutes unloaded (UL). (B) 15 min while carrying an infant dummy (2.73 kg) in arms (IA) (C) 15 min while babywearing an infant dummy in a structured baby carrier in an anterior position (BC)	-IA: increases the mechanical load on the knee and hip joints in the frontal plane by increasing joint moments. Arm carry increases the loading knee abduction moment by 8.7% and the loading knee extension moment by 16.7%. -BC: more similar to unloaded gait. -They conclude that during prolonged transport, babywearers may choose to use a baby carrier instead of carrying the baby in their arms.
Hyun & Ryew. (2018) [19]	N = 9 ♀ experienced in baby delivering (27.88 ± 2.71 years)	-1st and 2nd peak vertical force (PVF) -Medial-lateral and anteroposterior COPs -Extrapolated center of mass (XCoM). -Leg stiffness.	-GRF system and camera (4 units). -All participants wore 6 cm high heels. Randomly: (A) In normal conditions (no load). (B) Babywearing using a rear baby carrier.	-1st PVF: statistically significant differences according to the use of high heels and walking conditions, being higher when carrying a baby. -2nd PVF: statistically significant differences according to walking conditions. -Medial-lateral COP: showed statistically significant differences according to heel height and walking conditions. -Anteroposterior COP: showed no statistically significant differences according to heel shoe use and walking conditions during gait. -XCoM: showed statistically significant differences in the case of wearing heels. -Leg stiffness: showed statistically significant differences in the case of wearing heeled shoes and walking conditions.
Fista et al. (2019) [4]	N = 12 ♀ (between 20 and 22 years)	Posture (horizontal cranial, cranio-vertebral, and sagittal angles). -HR. -Borg discomfort (shoulders, neck, back, and trunk). -ABC scale (balance, self-confidence not to lose balance).	-Walking on a treadmill for 10 min. 3 different types of baby carriers: (A) SSC harness (B) Jarik (C) Ring sling	Posture: -All types of baby carriers produced a mean value of 49–52° in the sagittal angle. -In the horizontal cranial angle, the SSC-type sling provided the best change in posture. ABC scale: -According to the results of the ABC scale questionnaire, the SSC sling provided a higher level of confidence than the Jarik and the ring sling. Pain: The ring sling and the Jarik produced more pain in the left shoulder than the SSC sling.
Mannen et al. (2020) [11]	N = 10♀ healthy nulliparous (27.4 ± 4.1 years)	-Pain: VAS -COP: 2 × 40 × 40 cm force plates during testing at 1000 Hz.	Stand still for 15 min on a force platform (COP) and VAS after exposure in each of the following conditions: (A) No load (B) Carrying a dummy in arms (C) Babywearing a dummy	Pain: -30% reported pain during unloading and carrying -50% reported pain when carrying in arms COP -Participants shifted their weight more frequently, spent more time in an asymmetrical position, and had greater areas of swinging in arm carry. -In the comparison between pain and non-pain sufferers, pain sufferers remained more stationary in all conditions; although the baby carrier caused pain participants to shift their weight more often, this was a positive change.
Havens et al. (2020) [17]	N = 10 ♀ healthy nulliparous (27.4 ± 4.1 years)	-Motion capture based on movements -Force platform. -Kinematics: (step length, step time, support time, gait speed, and step width). -GRF	Assess how they walk and bend down to pick up an object: (A) Without weight (B) In arms (C) Carrying in a baby carrier	Gait: -Carrying in arms: greater vertical GRF and momentum, and braking force compared to non-weight bearing. Greater back extension. -Significant but small differences (<5°) between conditions were found in lower limb kinematics. -2.2% greater stride length in ‘weightless’ compared to ‘in arms’. Recovery: -The majority of individuals performed the squat technique to retrieve the object. -There were significant differences in lower limb kinematics at squat depth between right and left legs. There were no differences in timing between the downward and upward phases of the squat between conditions.
Wall-Scheffler et al. (2007) [9]	N = 6 ♀ of reproductive age (20.5 ± 0.8 years)	-VO2max -Anthropometry: Lower limbs, mass, and height. -Kinematics: Stride length and contact time	-4 sessions of treadmill walking of 15 min: (A) Control: weight on waist. (B) Control 2: weight on belt and no braking. (C) Carrying dummy in arms (D) Carrying the baby carrier dummy in anterior position	Anthropometry: -Bitrochanteric width correlates significantly with stride length and contact time under the most normal level. Energy cost: -Significant differences between A and B; B and C; A and C; C and D. Most interestingly, the average increase in cost of carrying a baby in arms versus babywearing in a sling is 16% (ranging from 13% to 25% increase). Kinematics: -Normalized stride lengths are shorter during carrying in arms than during the other experimental conditions.
Ohashi et al. (2018) [20]	N = 14 healthy young adults	-Heart rate (HR) -Oxygen uptake (V4 O2) -Ventilation/minute (V4 E) -Tidal volume (TV) -Respiratory exchange rate (R) -Respiratory rate (RR) -Last-minute values for each degree were averaged.	-Individualized walking speed at 30% of maximum oxygen uptake at 0% grade incline. -The test started at 0% grade walking on the treadmill and increased by 2% every 5 min up to 8%. The test was performed in a randomized fashion: (A) Front condition (F) (infant forward facing with lullaby belt). (B) Back condition (B) (infant backwards with lullaby belt).	-HR, V4 O2, V4 E, TV, R, and RR increased significantly with increasing grade in each condition (every 5 min). -There were no significant differences in interaction effects in HR, V4 O2, V4 E, TV, R, and RR. -No significant differences were found in the interactions between obliquity, condition, and sex for any of the items.
Chen et al. (2019) [16]	N = 10 ♀ and 10 males (24.0 ± 2.5)	-MAWC -%HR = (HR work − HR rest) × 100% -Perceived exertion (Borg’s CR-10 scale) in the whole body, neck, right shoulder, left shoulder, upper back, middle back, lower back, and abdomen	Eight transport tasks were performed. HR and MAWC were measured during each task. At the completion of each task, the Borg CR-10 was run. Type of carrier: (A) Padded shoulder straps and belt, with waist and abdomen support. (B) Two-part backpack, which distributes the weight. Carrying time: 1 h and 4 h Type of carrying: front and back	MAWC, obtained *p* < 0.05 in: -Sex: males (16.9 kg) vs. females (13.2 kg). -Rear carry (15.4 kg) vs. front carry (14.7 kg). -Type of carry: A = 15.6 kg vs. A = 14.5 kg. -Time: 1 h (16.2 kg) vs. 4 h (11.7 kg). %FC: carrying 1 h (19.3%) vs. 4 h (14.6%). *p* < 0.01 Perceived effort -Whole body: *p* < 0.05 in time (4.6 in 1 h vs. 4.0 in 4 h). -Mid back: *p* < 0.05 being higher in women (3.2) than in men (2.3). -Lower back: women (4.7) higher than men (3.5) (*p* < 0.01); front carrying (4.5) higher than back carrying (3.6) (*p* < 0.01); 1 h carrying (4.5) higher than 4 h carrying (3.6) (*p* < 0.01) and type A (4.5) higher than type B (3.7) (*p* < 0.05).
Atthawuttikul & Khongkharat (2021) [15]	Older people	-Questionnaire -Maximum load/transporter.	-The three 3D models were imported into force simulation software along with Earth gravity constant = ~9600 Nm/s. Stiffness = ~0.40 MPa. Flexibility = ~0.26 MPa. Durability = ~1.6 MPa. against impact force.	-Model D3 was able to withstand the highest load. -Model D1 supported the lowest load. -The four main factors in the ergonomic design for older people were the following: (1) the posture of their shoulders and hips while using the carrier. (2) the seat and backrest areas as load points of the baby carrier. (3) the load points on their body. (4) the type of baby carrier.

ABC Scale: Activities-Specific Balanced Confidence Scale; BC: baby carrying; cm: centimeters; COP: center of pressure; GRF: ground reaction force; h: hour; HR: heart rate; Hz: hertz; IA: in arms; kg: kilograms; km: kilometers; L: loaded; LBC-B: Loaded with baby carrier on the back; LBC-F: Loaded with baby carrier in front; LBC-S: Loaded with baby carrier on the side; M: Moment of the force plate; min: minutes; MAWC: maximum acceptable weight of carrying; MPa: megapascals; MVC: maximum voluntary contraction; Nm/s: newton per meter/second; R: Respiratory exchange rate; RR: respiratory rate; SC: shoulder carry; SSC: Soft Structured Carrier; SWC: Standing without load; Temp: temperature; TV: tidal volume; UL: unloaded; V4 E: ventilation/minute; V4 O2: oxygen uptake; VAS: visual analogue scale; VO2max: Maximum Oxygen Consumption; XCoM: Extrapolated center of mass; ♀: female.

## Data Availability

No new data were created or analyzed in this study.

## Abbreviations

The following abbreviations are used in this manuscript:

EMG	Electromyography
PRISMA	Preferred Reporting Items for Systematic reviews and MetaAnalyses
WOS	Web of Science
PICO	Patient, Intervention, Comparison, Outcome
MESH	Medical Subject Heading
JBI	Joanna Briggs Institute Critical Appraisal Checklist for Analytical Cross-Sectional Studies
LBC-B	Loaded with baby carrier on the back
LBC-F	Loaded with baby carrier in front
PVF	Peak Vertical Force
COP	Center of pressure
HR	Heart Rate
SSC	Soft Structured Carrier

## References

[1] Williams L.R., Grisham L.M., Gebler-Wolfe M., Kelsch K., Bedrick A., Bader M.Y. (2021). Nurse Perceptions of Babywearing for Neonates with Neonatal Abstinence Syndrome in the Neonatal Intensive Care Unit. Adv. Neonatal. Care..

[2] Siddicky S.F., Bumpass D.B., Krishnan A., Tackett S.A., McCarthy R.E., Mannen E.M. (2020). Positioning and baby devices impact infant spinal muscle activity. J. Biomech..

[3] López Acuña E.S., Salmerón Ruiz M.A. (2014). El porteo ergonómico; Ergonomic babywearing. Pediatr. Integral..

[4] Fista B., Widyanti A., Muslim K., Salma S.A. (2019). Evaluation of Baby Carriers in Indonesia: Physiological and Biomechanical Approach. IOP Conf. Ser. Mater. Sci. Eng..

[5] Fagundes F., Teixeira F., Camargo L., Mochizuki L., Amorim C., Soares R. (2016). Análise eletromiográfica de músculos da coluna vertebral durante a marcha em diferentes formas de carregar o bebê; Electromyographic analysis of the spinal muscles during the gait in different ways to carry a baby. Conscientiae Saúde.

[6] Yuk G.C., Park R.J., Lee H.Y., Lee M.H., Lee J.H., Kuk J.S., Jang J.-S. (2010). The Effects of Baby Carrier and Sling in Muscle Activation of Trunk, Low Extremity and Foot Pressure. J. Korean Soc. Phys. Med..

[7] Azaman A., Isa N.M., Dzahir M., Xiang K.K. (2017). Effects of baby carrier on wearer’s posture stability. J. Mech. Eng..

[8] Williams L., Standifird T., Madsen M. (2019). Effects of infant transportation on lower extremity joint moments: Baby carrier versus carrying in-arms. Gait Posture.

[9] Wall-Scheffler C., Geiger K., Steudel-Numbers K. (2007). Infant carrying: The role of increased locomotory costs in early tool development. Am. J. Phys. Anthropol..

[10] Ojukwu C.P., Okafor C.J., Chukwu S.C., Anekwu E.M., Okemuo A.J. (2019). Evaluation of selected cardiopulmonary and perceived exertion responses to four infant carrying methods utilised by African Mothers. J. Obstet Gynaecol..

[11] Mannen E., Havens K., Kahney A., Nelson-Wong E. (2020). Baby-Carrying Method Impacts Caregiver Postural Sway and Pain During Prolonged Standing. J. Womens Health.

[12] Page M.J., McKenzie J.E., Bossuyt P.M., Boutron I., Hoffmann T.C., Mulrow C.D., Shamseer L., Tetzlaff J.M., Akl E.A., Brennan S.E. (2021). The PRISMA 2020 statement: An updated guideline for reporting systematic reviews. BMJ.

[13] Joanna Briggs Institute (2020). Checklist for Analytical Cross Sectional Studies Critical Appraisal. https://jbi.global/sites/default/files/2020-08/Checklist_for_Analytical_Cross_Sectional_Studies.pdf.

[14] Leno-Durán E., Micha-Mabale M., García-Pérez M., Bueno-Cavanillas A., Barrios-Rodríguez R., Requena P. (2023). Influencia de la dieta en el riesgo de infección y de gravedad de la COVID-19: Una revisión sistemática; Influence of diet in COVID-19 infection and severity risk: A systematic review. Nutr. Hosp..

[15] Atthawuttikul A., Khongkharat S. (2021). Factors in Ergonomic Design of 6-to-18-month Baby Carriers for Elderly People. Pertanika J. Sci..

[16] Chen T.H., Kao Y.Y., Wang M.J.J., Goonetilleke R.S., Karwowski W. (2019). The Psychophysical Evaluations of Baby Carriers. Advances in Physical Ergonomics & Human Factors.

[17] Havens K.L., Severin A.C., Bumpass D.B., Mannen E.M. (2020). Infant carrying method impacts caregiver posture and loading during gait and item retrieval. Gait Posture.

[18] Lee H., Hong K. (2018). Type and wearing method-dependent COP and muscle fatigue measurement of baby carriers for the development of smart baby carriers. Int. J. Cloth. Sci. Technol..

[19] Hyun S.H., Ryew C.C. (2018). Effect of lower limb kinetic on carrying infant by hip seat carrier during high heel gait. J. Exerc. Rehabil..

[20] Ohashi K., Ono K., Kawate Y., Watase R., Ishikawa A. (2018). Respiratory response during upslope walking with different ways to carry a baby using baby carrier. Jpn. J. Phys. Fit. Sports Med..

[21] Wu C.Y., Huang H.R., Wang M.J. (2017). Baby carriers: A comparison of traditional sling and front-worn, rear-facing harness carriers. Ergonomics.

[22] Han J.H., Rankin L., Lee H., Feng D., Grisham L.M., Benfield R. (2024). Infant and parent heart rates during a babywearing procedure: Evidence for autonomic coregulation. Infant Behav. Dev..

[23] Nadeem A., Faisal S., Waqas S. (2024). Association Between Infant Carrying Methods, Duration, And Trunk Position Among Nursing Mothers of Lahore. J. Riphah. Coll. Rehabili. Sci..

